# Association Analysis between Cognitive Function Score and Inner Macular Thickness/Visual Field Sensitivity in Glaucoma Patients

**DOI:** 10.3390/jcm13175086

**Published:** 2024-08-27

**Authors:** Soichiro Shimomine, Suguru Kubota, Yoichi Kadoh, Masaki Tanito

**Affiliations:** Department of Ophthalmology, Shimane University Faculty of Medicine, Izumo 693-8501, Japanykadoh@med.shimane-u.ac.jp (Y.K.)

**Keywords:** glaucoma, cognitive impairment, Mini-Cog, OCT, visual field, VF reliability

## Abstract

(1) **Background**: Previous research has investigated the relationship between cognitive impairment, optical coherence tomography (OCT), visual fields (VF), and VF reliability in smaller patient samples using various cognitive assessment tools. This study analyzed the relationship between cognitive function scores using the Mini-Cog test and inner macular thickness (IMT) and VF sensitivity in glaucoma patients. (2) **Methods**: A retrospective analysis was conducted on 984 patients with 1897 eyes. Assessments included age, sex, intraocular pressure (IOP), and Mini-Cog test scores. Abnormal Mini-Cog scores were observed in 89 patients (9%). Using a mixed-effects model adjusted for background factors, the association between Mini-Cog scores and IMT, parafoveal (PF)-IMT, mean deviation (MD), pattern standard deviation, fixation losses (FL), false negatives (FN), and false positives (FP) was analyzed. (3) **Results**: Abnormal Mini-Cog scores (≤2) were associated with thinning of the IMT and PF-IMT, worse MDs, and higher FN and FP rates but not with PSD or FL. (4) **Conclusions**: Glaucoma patients with low cognitive function scores exhibited more advanced glaucoma-related changes in VF testing and morphological tests. Further longitudinal studies are needed to explore the relationship between glaucoma and cognitive impairment.

## 1. Introduction

Glaucoma is characterized by visual field (VF) defects due to optic neuropathy with progressive retinal ganglion cell degeneration and retinal nerve fiber layer (RNFL) thinning [1,2,3,4,5]. Optic nerve atrophy attributed to cerebral cortex damage has also been observed in early cognitive impairment [6]. The possibility of a connection between glaucoma and cognitive impairment has been implied for a long time [7]. Previous studies have reported that peripapillary RNFL thickness and/or macular ganglion cell-inner plexiform layer (GC-IPL) thickness were found to be significantly reduced in patients with Alzheimer’s disease (AD) and mild cognitive impairment (MCI) [8,9]. Lee’s group reported a potential association between retinal findings observed on optical coherence tomography (OCT) and cognitive impairment [7], indicating a possible link between OCT measurements and cognitive function in glaucoma patients. Primary open-angle glaucoma (PG) eyes had smaller mean lamina cribrosa thicknesses (LCTs) compared to control eyes [10]. Lower Mini-Mental State Examination (MMSE) scores were significantly associated with smaller mean LCTs, particularly affecting visuospatial function and visual memory [10]. Lower MMSE scores in PG and normal-tension glaucoma (NTG) patients compared to healthy controls have been reported [11]. These findings suggest that OCT could be a valuable tool in monitoring cognitive function in glaucoma patients, warranting further research to establish its utility as a biomarker for cognitive impairment.

In a study conducted by Diniz-Filho et al., global cognitive decline, as measured by the Montreal Cognitive Assessment (MoCA) test, was associated with VF variability in patients with glaucoma and those suspected of having the disease, with the patients being followed-up over time [12]. Our research team identified that cognitive impairment may affect the consistency of VF testing in patients with glaucoma, emphasizing the necessity of incorporating cognitive function assessments into glaucoma management [13]. Therefore, when a marked rise in VF variability due to cognitive decline is observed, clinicians might consider increasing the frequency of tests to achieve more accurate longitudinal measurements. Another approach could be to place greater emphasis on alternative tests for monitoring progression, such as structural imaging of the optic nerve, nerve fiber layer, or macular regions [12]. Moreover, cognitive impairment can influence patients’ performances on both structural and functional glaucoma assessments.

Prior studies have explored the connection between cognitive impairment, OCT, VF, and VF reliability in smaller patient cohorts utilizing various cognitive assessment tools, including the MMSE (137 patients, no ocular data) [4], MoCA test (61 patients, 95 eyes) [14], and clock drawing test (60 patients, 113 eyes) [15]. In our study, we examined this relationship using the Mini-Cog test, which involved the largest dataset to date, encompassing nearly 1000 subjects. To the best of our knowledge, this is the first study that has explored the association between the Mini-Cog test and retinal structural and functional measures and VF reliability in glaucoma patients simultaneously. Here, we report the analysis of the relationship between cognitive function scores and macular inner retinal layer thickness/visual field sensitivity and reliability in patients with glaucoma.

## 2. Materials and Methods

### 2.1. Study Design and Subjects

This retrospective study complied with the principles outlined in the Declaration of Helsinki and received approval from the Institutional Review Board (IRB) of Shimane University Hospital (study no. 20220616-1, issued on 3 April 2023). The IRB determined that written informed consent from each patient was not necessary for publication. Instead, the study protocol was publicized at the participating institutions to inform participants about the research.

Subjects were selected from individuals visiting the glaucoma outpatient clinic at Shimane University Hospital, Japan, between March 2020 and March 2023. All the patients who took a cognitive function test with the Mini-Cog underwent a fundus examination with optical coherence tomography (RS-3000 Advance II, Nidek, Gamagori, Japan) and received a VF test with the Central 30-2 program of the Humphrey Visual Field Analyzer (Carl Zeiss Meditec, Dublin, CA, USA) using the SITA-standard algorithm. The study encompassed all patients who attended the glaucoma outpatient clinic and fulfilled the specified criteria, thereby incorporating individuals with different types of glaucoma as well as those identified as glaucoma suspects. As a result, 1897 eyes of 984 subjects were included in the study. Most subjects were Japanese.

The diagnostic evaluation of glaucoma rests on the IOP, ocular function, and morphology [16]. PG was diagnosed by the presence of open iridocorneal angles, typical signs of glaucomatous optic neuropathy such as expanded optic disc cups or localized thinning of the neuroretinal rim, corresponding visual field impairments by the central 30-2 or 10-2 programs in at least one eye, and the absence of secondary glaucoma bilaterally. Exfoliation glaucoma (EG) was diagnosed by identifying an open iridocorneal angle along with visible pseudoexfoliation material deposits on the anterior capsule or pupillary edge in at least one eye. In this study, exfoliation syndrome without IOP elevation and/or VF defects was also included in the EG group. All diagnoses were made by a single author (M.T.). We examined the medical charts retrospectively and collected the data on age at Mini-Cog testing, sex, best-corrected visual acuity (BCVA), spherical equivalent refractive error (SERE), intraocular pressure (IOP), number of glaucoma medications, Mini-Cog scores, the three types of glaucoma (PG, EG, and others), morphological parameters including inner macular thickness (IMT) and parafoveal inner macular thickness (PF-IMT), and VF-derived parameters including mean deviation (MD), pattern standard deviation (PSD), and rates of fixation losses (FL), false negatives (FN), and false positives (FP). IOP was measured by a Goldmann applanation tonometer (GAT). Decimal BCVA values were transformed into the logarithm of the minimum angle of resolution (LogMAR). All ophthalmological data were taken from the date closest to the Mini-Cog test date.

### 2.2. Measurements of Mini-Cog Score

In this study, the Mini-Cog test was used to assess cognitive function. The Mini-Cog test, a straightforward cognitive screening tool, involves three-item word recall and a clock drawing test [17]. Overall Mini-Cog scores can range from 0 to 5 points, with word recall contributing 0 to 3 points and the clock drawing test adding either 0 or 2 points. The Mini-Cog test results are assessed by an algorithm as “possibly impaired (score ≤ 2)” or “probably normal (score ≥ 3)”, and the MMSE, with a cut-off point of 25, shows similar sensitivity (76% vs. 79%) and specificity (89% vs. 88%) for dementia [17]. Our hospital routinely evaluates cognitive function in patients with glaucoma. The Mini-Cog test is usually performed by skilled ophthalmic nurses during patients’ initial visits and when providing medication instructions regarding eye drops.

### 2.3. Measurements of Morphological Parameters

Macular scans were recorded in a 9 mm × 9 mm area with 128 vertical scans (512 pixels/scan). The inner macular layers were determined to be between the inner limiting membrane and the inner plexiform/inner nuclear layer border as delineated by the OCT device’s software (version 2.22.00). The IMT was defined as the average thickness of the inner macular layers in the 9 mm diameter area centered at the fovea. The PF-IMT was defined as the average inner macular thickness in the donut-shaped areas with a radius of 1.5 mm to 4.5 mm centered at the fovea. The measurements closest to the date of the Mini-Cog test were adopted. Referring to the signal quality index and signal strength index, if the image closest to the Mini-Cog test date was unclear, the image data from the next closest date were used. All measurements were performed by skilled ophthalmic examiners.

### 2.4. Statistical Analysis

For statistical purposes, the subjects were categorized into 895 Mini-Cog normal (score ≥ 3) subjects and 89 Mini-Cog abnormal (score ≤ 2) subjects. For eye-based analyses, 1727 eyes and 170 eyes were categorized into the respective groups. The data were presented as means ± standard deviations (SDs) with 95% confidence intervals for continuous variables, and as numbers and percentages for categorical variables. The potential relationship between normal and abnormal Mini-Cog scores was assessed using an unpaired *t*-test for the continuous variables and a Fisher’s exact test or Pearson’s chi-square test for the categorical variables. In these evaluations, age, sex, and Mini-Cog scores were analyzed on a subject basis, while SERE, number of glaucoma medications, IOP, the three glaucoma types, morphological parameters, and VF-derived parameters were analyzed on an eye basis. These associations were further examined through a multivariable analysis using a mixed-effects regression model. In this model, the eye-based analysis was conducted, and any bias due to the inclusion of both eyes from a single subject was adjusted by incorporating the subject’s identification number as a random effect. All statistical analyses were carried out using JMP Pro statistical software version 16.1.0 (SAS Institute, Inc., Cary, NC, USA). A *p*-value of less than 0.05 was deemed statistically significant.

## 3. Results

The demographic details of the subjects, including age, sex, Mini-Cog scores, SERE, number of glaucoma medications, IOP, the three types of glaucoma, IMT, PF-IMT, MD, PSD, FL, FN, and FP, are summarized in Table 1.

Table 2 compares the groups based on the normal and abnormal total Mini-Cog scores. With a cutoff value of a total Mini-Cog score of ≤2, the suspected prevalence of cognitive impairment was found to be 9.0% (89 out of 984) among the patients visiting our glaucoma clinic. Compared to the normal Mini-Cog score group, the abnormal Mini-Cog score group exhibited several significant differences, as follows: older age (*p* < 0.0001), worse BCVA (*p* < 0.0001), less myopic SERE (*p* < 0.0001), fewer glaucoma medications (*p* < 0.0001), lower IOP (*p* = 0.002), and a lower prevalence of PG (*p* < 0.0001), with sex being comparable between the two groups. Regarding the morphological parameters, the abnormal Mini-Cog score group had a smaller PF-IMT value (*p* = 0.006) than the normal Mini-Cog score group, while IMT (*p* = 0.06) values were equivalent in both groups. Considering the VF-derived parameters, the abnormal Mini-Cog score group had worse MD (*p* = 0.007) and higher FN (*p* = 0.001) values, while the PSD (*p* = 0.2), FL (*p* = 0.5), and FP (*p* = 0.09) values were equivalent in both groups.

Table 3 displays the results from the multivariable analysis model (Model 1) that investigated the factors linked to the morphological parameters. After adjusting for various background variables, an abnormal Mini-Cog score was found to be associated with reductions in IMT (*p* = 0.01) and PF-IMT (*p* = 0.005). Additionally, aging was associated with a decreasing PF-IMT (*p* = 0.01). A less myopic SERE exhibited an association with a thicker IMT (*p* < 0.0001) and PF-IMT (*p* < 0.0001), and a larger number of glaucoma medications was correlated with a thinner IMT (*p* < 0.0001) and PF-IMT (*p* < 0.0001). Lastly, compared with PG, EG was associated with a thinner IMT (*p* = 0.02), while other types of glaucoma were associated with thicker IMTs (*p* = 0.001).

Table 4 details the results from the multivariable analysis model (Model 2) examining the factors associated with the VF-derived visual functions. After adjusting for various background variables, an abnormal Mini-Cog score was associated with a worse MD (*p* = 0.005) but did not show a significant relationship with PSD (*p* = 0.7). A less myopic SERE was linked to a better MD (*p* = 0.006) and a lower PSD (*p* = 0.007). A greater number of glaucoma medications was associated with a worse MD (*p* < 0.0001) and a higher PSD (*p* < 0.0001). Additionally, a higher IOP was connected to a better MD (*p* = 0.0009) and a lower PSD (*p* < 0.0001). Compared to PG, EG had a worse MD (*p* = 0.002), while other glaucoma types were associated with lower PSDs (*p* = 0.003).

Table 5 outlines the results from the multivariable analysis model (Model 3) that investigated the factors linked to the VF reliability parameters. After adjusting for various background variables, an abnormal Mini-Cog score was associated with an increased FN (*p* = 0.007) and a higher FP (*p* = 0.03). Additionally, aging was correlated with an elevated FL (*p* = 0.02) and an increased FN (*p* < 0.0001).

## 4. Discussion

This study sought to assess the impact of cognitive function on both morphological and functional changes in glaucoma patients. Our results suggested that, based on the Mini-Cog test, 9% of the patients attending the glaucoma clinic were suspected of having cognitive impairment. After adjusting for background variables using mixed-effects regression models, abnormalities in the Mini-Cog scores were linked to thinning of both the IMT and PF-IMT, a worse MD, and higher FN and FP rates, while no association was found with PSD and FL rates. Apart from causality relationships, our results clearly suggest that glaucoma patients with low cognitive function scores show more advanced glaucoma-related changes in both visual field testing and morphological testing. Our results also showed that an abnormal Mini-Cog score was connected to a lower reliability of the VF testing.

The Mini-Cog is a brief cognitive screening tool that focuses on memory and executive function, making it particularly useful for rapid assessment in clinical settings. Compared to the more comprehensive MMSE and MoCA tests, the Mini-Cog is ideal for situations where a quick evaluation is required, though it may lack the detailed diagnostic capabilities of the other tools. Regarding the OCT-derived morphology parameters, the IMT values were equivalent in the normal and abnormal total Mini-Cog score groups (Table 2). However, after adjusting for background parameters, the correlation between lower cognitive function scores and thinner IMTs and PF-IMTs was revealed (Table 3). These findings were consistent with a report by Kesler et al., which showed that RNFL thinning was more prominent in subjects with cognitive impairments such as AD [18]. McCoskey et al. found no significant differences in the total MoCA scores between the PG cases and healthy controls, nor was there any significant association between the RNFL thickness and the total MoCA score [4]. In addition to the differences in the methods used to assess cognitive function, differences in the detection power derived from the differences in the sample sizes might explain this discrepancy. In a meta-analysis that evaluated various cognitive impairment screening methods, the prevalence of MCI among glaucoma patients ranged from 12.3% to 90.2%, while the prevalence of dementia was reported to be between 2.5% and 3.3% [19]. In contrast, our study observed a cognitive impairment prevalence of 9% (Table 2), which differed from previous findings. The Mini-Cog test is generally not suited for detecting advanced dementia, as it primarily targets MCI and mild-to-moderate dementia. This suggests that the choice of screening test could explain the differences in the observed prevalence [13].

Our findings indicated that an abnormal Mini-Cog score was associated with a worse MD (Table 4). The neural changes linked to cognitive impairment in glaucoma patients may result from the reduction in sensory input caused by VF defects. Two population-based studies, one conducted in the United States (*n* = 2520) and another in Singapore (*n* = 2478), have reported significant correlations between visual impairment and lower cognitive test scores using the MMSE and the abbreviated mental test, respectively [20,21]. Additionally, the prospective data from the Women’s Health Initiative cohort, with a mean follow-up of 3.8 years (*n* = 1061), revealed that individuals with visual impairment had a five- to six-fold higher risk of developing dementia and mild cognitive impairment [22]. These earlier studies suggested that visual impairment could lead to cognitive decline. Similarly, McCoskey’s group found that more severe glaucoma, characterized by greater VF loss, was linked to poorer cognitive function as measured by the MoCA [4]. However, they also noted that diminished vision and reduced VF could directly impact a patient’s performance on MoCA testing, particularly in sections requiring vision for reading and writing [4]. Furthermore, previous research has documented the influence of aging on VF sensitivity [23,24,25]. These complexities make it challenging to examine the potential relationship between VF loss in glaucoma and cognitive function.

We also explored the potential impact of cognitive impairment on the reliability of VF testing. Our findings suggested that a lower Mini-Cog score could compromise VF test reliability, as indicated by the association between an abnormal Mini-Cog score and higher FN and FP rates (Table 5). This result aligned with our previous report, which also demonstrated that, after adjusting for various background factors, an abnormal Mini-Cog score was linked to increased FN and FP rates but not to FL [13]. Although not definitive, the findings implied that FN may be more influenced by memory dysfunctions, as reflected in the word recall test, while FP might be more affected by non-memory cognitive impairments, such as executive and visuospatial difficulties, as indicated by the clock-drawing test [13]. Conversely, a study by Bastani et al. found a significant negative correlation between MoCA scores and both PSD and FN in their study population [14]. The MoCA is designed to assess attention, concentration, executive functions, memory, language, visuospatial skills, conceptual thinking, calculations, and orientation, thus consisting of more subdivided items than the Mini-Cog. Accordingly, these contradictory findings can be explained by the different items contained in each cognitive test. Diniz et al. observed an increase in VF variability over a 2.5-year follow-up period, during which they monitored the progression of cognitive impairment and its impact on VF indices [12]. Given that both glaucoma and cognitive impairment are progressive conditions, further longitudinal studies are needed to explore how cognitive impairment and VF reliability evolve in glaucoma patients. The fact that aging and cognitive decline reduce the reliability of VF testing indicates the need to develop objective and multifaceted visual function testing methods that are not influenced by these factors in the future. In cases of unreliable VF testing, OCT, as an objective measure, is advantageous. On the other hand, OCT is an effective test method for the diagnosis and follow-up of early glaucoma. In the elderly, the prevalence of advanced glaucoma is high, and clean OCT images are often not available due to the presence of cataracts and small pupils. Therefore, current diagnostic techniques often hamper diagnoses in the elderly and cognitively impaired.

This study had several limitations. Similar to other retrospective studies, it may be prone to patient selection bias. However, we included all patients presenting to the glaucoma outpatient clinic of our hospital, which might have helped to decrease selection bias. Due to the nature of a university hospital, a majority of the patients in this study were advanced cases or required surgical treatment. This should be taken into account when generalizing the results of this study. Furthermore, this study was carried out in a region of Japan with a predominantly older population, and so caution should be exercised when generalizing our findings to younger populations. Lower IOPs and a smaller number of glaucoma medications were found in the abnormal Mini-Cog score group (Table 2), and a higher IOP was associated with a better MD in the multiple regression model (Table 4). These findings might reflect that more advanced cases and/or older cases were treated with fewer eye drops and were less dependent on adherence (i.e., surgery). Therefore, the missing data on medication adherence and surgical history were a limitation of the study. Systemic factors such as hypertension, diabetes, cancer, and various neurodegenerative diseases may also affect glaucoma and cognitive function. The lack of data on these factors was also a limitation. Despite these limitations, we believe that the results of this study represent real-world outcomes shaped by a variety of pathological and background factors. Notably, our findings offer valuable insights into the connections between glaucoma, visual field testing, OCT, and cognitive function. Our results reflect that glaucoma and cognitive decline share aging as a common cause. However, due to the nature of a cross-sectional study, it is not possible to determine whether visual impairment due to glaucoma itself causes cognitive decline. Future large-scale, long-term longitudinal studies should be conducted to explore the causal relationship between glaucoma and cognitive impairment.

## 5. Conclusions

The associations between cognitive impairment and both retinal structural and functional measures, as well as VF reliability, were simultaneously explored in several types of glaucoma patients. The multivariable analyses revealed that a total Mini-Cog score of ≤2 was linked to a thinner IMT and PF-IMT, a worse MD, and higher FN and FP values. These results suggest that cognitive impairment may affect IMT, VF sensitivity, and VF reliability in glaucoma patients. The early detection and evaluation of cognitive impairment using tools like the Mini-Cog test could enhance the overall management of glaucoma patients.

## Figures and Tables

**Table 1:** Demographic data based on the subjects and their eyes.

Parameters	N or Mean ± SD	% or 95% CI
**Subjects**	984	
Age, year	70.4 ± 12.6	69.6, 71.2
Sex		
Male	522	53.0%
Female	462	47.0%
**Eyes**	1897	
BCVA, LogMAR	0.2 ± 0.5	0.2, 0.3
SERE, D	−2.0 ± 3.2	−2.2, −1.9
Number of glaucoma medications	2.6 ± 1.5	2.5, 2.7
IOP, mmHg	16.7 ± 6.6	16.4, 17.0
The type of glaucoma		
PG	1055	55.6%
EG	334	17.6%
Others	508	26.7%
Mini-Cog Scores		
≤2	178	9.1%
≥3	1790	91.0%
IMT, µm	30.2 ± 8.5	29.7, 30.6
PF-IMT, µm	84.5 ± 21.6	83.5, 85.5
MD, dB	−8.2 ± 6.3	−8.6, −7.8
PSD, dB	8.1 ± 4.6	7.9, 8.3
FL, %	10.1 ± 12.2	9.5, 10.7
FN, %	6.1 ± 8.6	5.6, 6.5
FP, %	2.3 ± 3.4	2.1, 2.5

Abbreviations: BCVA, best-corrected visual acuity; LogMAR, logarithm of the minimum angle of resolution; SERE, spherical equivalent refractive error; D, diopter; IOP, intraocular pressure; PG, primary open-angle glaucoma; EG, exfoliation glaucoma; IMT, inner-macular thickness; PF-IMT, parafoveal-inner macular thickness; MD, visual field mean deviation; PSD, visual field pattern standard deviation; FL, fixation losses; FN, false negative; FP, false positive; 95% CI: 95% confidence interval.

**Table 2:** Comparison between the groups stratified by Mini-Cog scores.

Parameters	Mini-Cog ≤ 2		Mini-Cog ≥ 3		*p*-Value
	N or Mean ± SD	% or 95% CI	N or Mean ± SD	% or 95% CI	
**Subjects**	89	9%	895	91%	
Age	80.5 ± 7.7	78.8, 82.1	69.4 ± 12.5	68.6, 70.3	<0.0001 **
Sex	M, 43	48%	M, 479	54%	0.3
Eyes	170	9%	1727	91%	
BCVA, LogMAR	0.5 ± 0.8	0.4, 0.6	0.2 ± 0.5	0.2, 0.2	<0.0001 **
SERE, D	−0.6 ± 1.7	−0.9, −0.4	−2.2 ± 3.3	−2.3, −2.0	<0.0001 **
Number of glaucoma medications	2.1 ± 1.3	1.9, 2.3	2.7 ± 1.5	2.6, 2.7	<0.0001 **
IOP, mmHg	15.2 ± 6.2	14.2, 16.1	16.8 ± 6.6	16.5, 17.1	0.002 **
The type of glaucoma					
PG	66	39%	989	57%	<0.0001 **
EG	47	28%	287	17%	
Others	57	34%	451	26%	
IMT, µm	67.0 ± 16.4	64.5, 69.5	69.5 ± 16.2	68.8, 70.3	0.06
PF-IMT, µm	80.2 ± 22.8	76.7, 83.7	84.9 ± 21.4	84.0, 85.9	0.006 **
MD, dB	−10.2 ± 6.5	−11.7, −8.7	−8.1 ± 6.2	−8.4, −7.7	0.007 **
PSD, dB	7.7 ± 3.9	7.0, 8.4	8.2 ± 4.6	7.9, 8.4	0.3
FL, %	10.7 ± 12.3	8.6, 12.9	10.0 ± 12.2	9.4, 10.7	0.5
FN, %	9.5 ± 11.1	7.3, 11.7	5.8 ± 8.3	5.3, 6.3	<0.0001 **
FP, %	2.8 ± 4.8	1.9, 3.6	2.2 ± 3.2	2.1, 2.4	0.09

The *p*-values were calculated by unpaired *t*-tests for the continuous data and by Fisher’s exact probability tests or chi-square tests for the categorical data. The ** denotes significant levels of 1% (*p* < 0.01). Abbreviations: BCVA, best-corrected visual acuity; LogMAR, logarithm of the minimum angle of resolution; SERE, spherical equivalent refractive error; D, diopter; IOP, intraocular pressure; PG, primary open-angle glaucoma; EG, exfoliation glaucoma; IMT, inner-macular thickness; PF-IMT, parafoveal-inner macular thickness; MD, visual field mean deviation; PSD, visual field pattern standard deviation; FL, fixation losses; FN, false negative; FP, false positive; 95% CI: 95% confidence interval.

**Table 3:** Multivariable analysis of the factors related to the morphological indices for Model 1.

Parameters	IMT				PF-IMT			
	Estimate	95% CI	*p*-Value	VIF	Estimate	95% CI	*p*-Value	VIF
Age, /year	−0.07	−0.15, 0.01	0.09	1.4	−0.14	−0.25, −0.03	0.01 *	1.4
Sex, F/M	−0.11	−0.96, 0.75	0.8	1.0	−0.05	−1.20, 1.09	0.9	1.0
SERE, D	1.02	0.73, 1.31	<0.0001 **	1.3	1.22	0.84, 1.60	<0.0001 **	1.3
Number of glaucoma medications	−2.30	−2.86, −1.74	<0.0001 **	1.1	−2.55	−3.30, −1.80	<0.0001 **	1.1
IOP, /mmHg	0.01	−0.11, 0.13	0.9	1.1	−0.12	−0.27, 0.04	0.2	1.1
EG/PG	−1.69	−3.09, −0.29	0.02 *	2.0	−1.46	−3.35, 0.44	0.1	2.0
Other/PG	2.24	0.90, 3.58	0.001 *	1.9	1.68	−0.12, 3.48	0.07	1.9
Mini-Cog score, ≤2/≥3	−3.95	−7.03, −0.87	0.01 *	1.1	−5.86	−9.97, −1.74	0.005 **	1.1

The *p*-values were calculated by a mixed effects regression model. The * and ** denote significant levels of 5% (*p* < 0.05) and 1% (*p* < 0.01), respectively. Abbreviations: IMT, inner-macular thickness; PF-IMT, parafoveal-inner macular thickness; 95% CI, 95% confidence interval; VIF, variance inflation factor; SERE, spherical equivalent refractive error; D, diopter; IOP, intraocular pressure; PG, primary open-angle glaucoma; EG, exfoliation glaucoma.

**Table 4:** Multivariable analysis of the factors related to the visual field indices for Model 2.

Parameters	MD				PSD			
	Estimate	95% CI	*p*-Value	VIF	Estimate	95% CI	*p*-Value	VIF
Age, /year	−0.03	−0.07, 0.01	0.1	1.4	−0.01	−0.03, 0.01	0.4	1.4
Sex, F/M	−0.10	−0.50, 0.30	0.6	1.0	0.02	−0.23, 0.27	0.9	1.0
SERE, D	0.19	0.06, 0.33	0.006 **	1.3	−0.12	−0.20, −0.03	0.007 **	1.3
Number of glaucoma medications	−1.17	−1.44, −0.90	<0.0001 **	1.1	0.90	0.73, 1.07	<0.0001 **	1.1
IOP, /mmHg	0.11	0.05, 0.18	0.0009 **	1.1	−0.08	−0.12, −0.05	<0.0001 **	1.1
EG/PG	−1.20	−1.93, −0.46	0.002 **	2.1	0.40	−0.03, 0.83	0.07	2.0
Other/PG	0.57	−0.08, 1.22	0.09	2.0	−0.60	−1.00, −0.05	0.003 **	1.9
Mini-Cog score, ≤2/≥3	−2.25	−3.84, −0.67	0.005 **	1.1	0.18	−0.73, 1.08	0.7	1.1

The *p*-values were calculated by a mixed effects regression model. The ** denotes a significant level of 1%. Abbreviations: MD, visual field mean deviation; PSD, visual field pattern standard deviation; 95% CI, 95% confidence interval; VIF, variance inflation factor; SERE, spherical equivalent refractive error; D, diopter; IOP, intraocular pressure; PG, primary open-angle glaucoma; EG, exfoliation glaucoma.

**Table 5:** Multivariable analysis of the factors related to the visual field reliability indices for Model 3.

Parameters	FL				FN				FP			
	Estimate	95% CI	*p*-Value	VIF	Estimate	95% CI	*p*-Value	VIF	Estimate	95% CI	*p*-Value	VIF
Age, /year	0.08	0.01, 0.15	0.02 *	1.4	0.10	0.05, 0.16	<0.0001 **	1.4	0.004	−0.02, 0.02	0.7	1.4
Sex, F/M	0.44	−0.30, 1.18	0.2	1.0	−0.001	−0.54, 0.54	0.1	1.0	0.2	−0.05, 0.38	0.1	1.0
SERE, D	0.07	−0.18, 0.32	0.6	1.3	0.05	−0.13, 0.24	0.6	1.3	−0.02	−0.09, 0.05	0.6	1.3
Number of glaucoma medications	−0.05	−0.55, 0.45	0.8	1.1	0.24	−0.13, 0.60	0.2	1.1	0.05	−0.09, 0.19	0.5	1.1
IOP, /mmHg	0.02	−0.08, 0.13	0.7	1.1	−0.02	−0.10, 0.06	0.6	1.1	0.004	−0.02, 0.03	0.8	1.1
EG/PG	0.61	−0.62, 1.85	0.3	2.0	0.71	−0.21, 1.64	0.1	2.0	−0.3	−0.63, 0.06	0.1	2.0
Other/PG	−0.39	−1.55, 0.77	0.5	1.9	−0.50	−1.35, 0.36	0.3	2.0	0.01	−0.32, 0.34	1.0	1.9
Mini-Cog score, ≤2/≥3	0.22	−2.47, 2.90	0.9	1.1	2.82	0.77, 4.87	0.007 **	1.1	0.9	0.10, 1.66	0.03 *	1.1

The *p*-values were calculated by a mixed effects regression model. The * and ** denote significant levels of 5% (*p* < 0.05) and 1% (*p* < 0.01), respectively. Abbreviations: FL, fixation losses; FN, false negative; FP, false positive; 95% CI, 95% confidence interval; VIF, variance inflation factor; SERE, spherical equivalent refractive error; D, diopter; IOP, intraocular pressure; PG, primary open-angle glaucoma; EG, exfoliation glaucoma.

## Data Availability

The dataset is available on request from the authors.
